# A case report of diagnosis of cat-scratch disease using metagenomic next-generation sequencing

**DOI:** 10.3389/fcimb.2023.1322651

**Published:** 2024-01-15

**Authors:** Tao Zhou, Yaqiu Zheng, Huizi Zhang, Yongfang Liu

**Affiliations:** ^1^ Department of Infectious Disease Department, The Third People’s Hospital of Chengdu, Chengdu, China; ^2^ Digestive Endoscopy Center, Public Health Clinical Center of Chengdu, Chengdu, China; ^3^ Department of Geriatric, Bishan Hospital of Chongqing Medical University, Chongqing, China

**Keywords:** cat-scratch disease (CSD), *Bartonella henselae*, hypo-immunity, metagenomic next-generation sequencing (mNGS), diagnosis, treatment

## Abstract

Cat-scratch disease (CSD) is an anthropozoonotic infection caused by *Bartonella henselae*, and it is one of the most common causes of lymph node infections in children and adolescents. *B. henselae*, belonging to the genus *Bartonella*, is a common human pathogen of human beings. CSD commonly develops as a result of cat scratches and bites or when injured skin comes into contact with cat saliva. The manifestation of CSD clinically differs for each patient based on their immune system. Individuals who have healthy immune systems generally manifest minimal clinical symptoms and do not necessitate any form of treatment. However, patients who have hypo-immunity require prompt medical attention due to the potential manifestation of severe symptoms that affect multiple systems of the body. Long latency and atypical clinical manifestations are characteristics of CSD. *Bartonella* isolation and identification are challenging procedures that require specialized equipment. There is no gold standard method for CSD diagnosis, and misdiagnosis and missed diagnosis rates are typically high. We present the case of a middle-aged male patient who developed fever, chills, anal distension, dizziness, and muscle pain for 10 days. The patient had a documented history of cat bites 1 month prior to the onset of symptoms. Following admission, he underwent an examination to determine superficial lymphadenopathy and hypoimmunity. Additionally, he had a fever during the disease. As the patient refused a needle biopsy of lymph nodes, metagenomic next-generation sequencing (mNGS) was employed and *B. henselae* was detected in the peripheral blood. The patient was diagnosed with CSD and treated with a combination of azithromycin and doxycycline. The fever symptoms were alleviated, and the patient was ultimately discharged. As a result of this case, we suggest that mNGS be used as a crucial supplementary diagnostic tool for individuals with compromised immune systems who may have CSD, especially when conventional diagnostic methods are inconclusive.

## 1 Background

Cat-scratch disease (CSD) is a widespread infectious disease, particularly prevalent in countries characterized by warm and humid climates during the spring and winter seasons (Schutze, 2000). CSD can be attributed to the cyclical breeding patterns of cats (Windsor, 2001). It is a common cause of lymph node infections among children and adolescents (Margileth, 2000). While CSD was first reported in 1950, its causative agent was not determined until 1992. Serology and microbiology studies confirmed that *Bartonella henselae* is related to CSD (Chomel, 2000). *B. henselae* mostly resides in the oropharynx of felines and can be transmitted to human beings by cat bites, scratches, saliva droplets, or licking. Cats serve as primary vectors of fleas, which play a crucial role in transmitting diseases between cats (Chomel, 2000). *Ixodes ricinus* has been identified as a new transmission vector of *B. henselae* (Cotté et al., 2008).

CSD, often referred to as benign lymphoreticulocytosis, primarily presents diverse clinical presentations that are dependent upon the patient’s immune status. Individuals with healthy immune systems exhibit classic CSD symptoms, which include the appearance of a small painless red bump or pus-filled lesion at the site of infection within 2 weeks. Additionally, they may experience swelling of the lymph nodes in the area where the infection occurred on the same side of the body, typically occurring between 1-3 weeks after the initial infection. Patients with hypo-immunity, on the other hand, exhibit atypical CSD caused by infection of organs and tissues (Juan and Gan, 2011) outside the lymph nodes through the lymphatic or bloodstream flow (Florin et al., 2008). CSD, in this case, may result in ocular (Johnson, 2020; Tey et al., 2020; Hong et al., 2023), neurologic (Florin et al., 2008; Juan and Gan, 2011), musculoskeletal (Maman et al., 2007; Dornbos et al., 2016), skin (Florin et al., 2008), and breast involvement (Gamblin et al., 2005) as well as other manifestation and syndromes (e.g., pneumonitis, pleural effusion, idiopathic thrombocytopenic purpura, anaphylactoid purpura, erythema multiforme, hypercalcemia, glomerulonephritis, and myocarditis).

CSD has a prolonged latency period and presents with diverse clinical signs. Disseminated CSD may be detected in individuals with compromised immune systems affecting several systems or in environments that pose a hazard to life. Therefore, timely detection of CSD is highly important. Metagenomic next-generation sequencing (mNGS) offers substantial advantages in rapidly determining infections and can surve as a supplementary technique for diagnosing CSD. A case of CSD diagnosed by mNGS is reported as follows:

## 2 Case study

**Figure 1:**
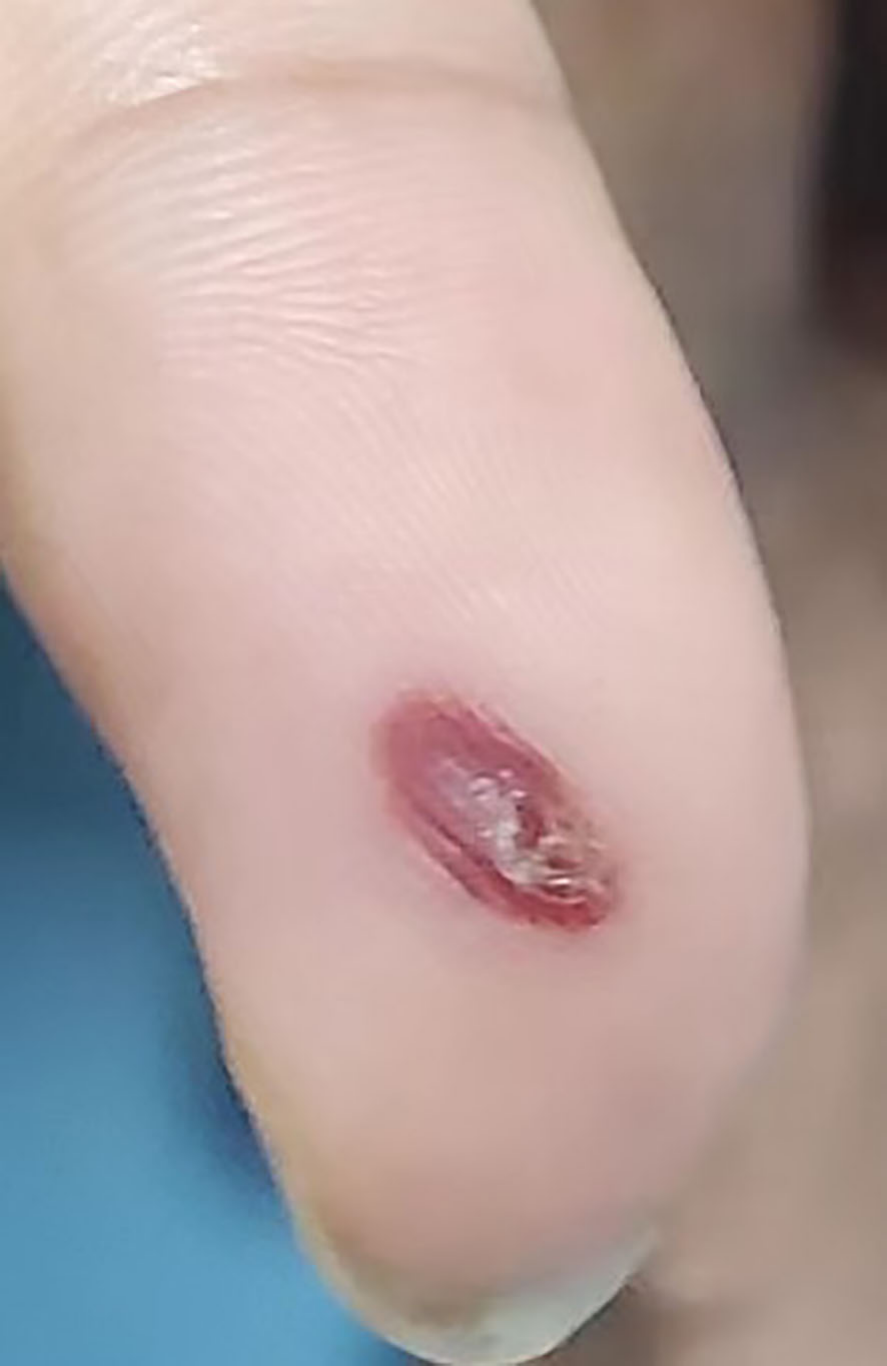
Primary skin lesions: Dark red erythema on the fingertips of the right hand, which did not fade under pressure, and partial breakage of the epidermis with desquamation.

**Figure 2:**
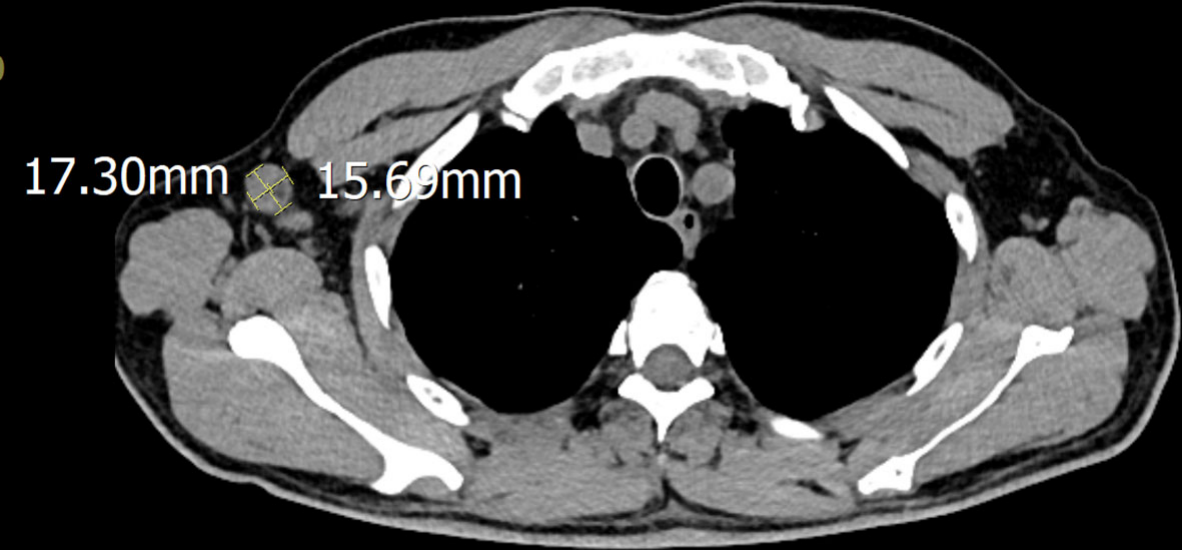
Lymph nodes in the right axilla under CT examination.

A 51-year-old male was admitted to the hospital after 10 days of fever with no obvious cause; the self-tested temperature was 38°C, and chills, anal distension, dizziness, muscle pain, and other discomforts accompanied with the fever. The patient visited the anorectal outpatient support and received medication in the form of an anal plug (specific details were undisclosed). While the anal distension was relieved, the patient still had a fever, and his body temperature fluctuated between 37°C and 37.5°C. Therefore, he received medical intervention at the hospital. The patient had chronic viral hepatitis B and had been treated with interferon-α for nearly 2 years at the time of admission. He has been smoking for 6 years, 10 cigarettes per day, and drinking alcohol for 6 years, with an average of 2 g of liquor per day. He showed no other chronic diseases, infectious diseases, family diseases, allergy history, surgical history, blood transfusion history, trauma history or mental illness. The dynamics within his family are characterized by harmony, and he experiences success in his professional endeavors. He has not visited locations affected by epidemics or regions with a high prevalence of infection diseases. He has no previous history of drug use or involvement in prostitution. Further interrogation revealed a history of cat bites a month prior to admission; the site of the bite was the tip of the right ring finger. Admission examination revealed skin lesions on the tip of the right ring finger (see **Figure 1**). Enlarged lymph nodes were detected in the right axilla, which was tender to touch, firm, and not adherent to the surrounding area (**Figure 2** shows the lymph nodes in the right axilla under CT examination).

The physical examination revealed that the cat bite was located on the tip of the right ring finger, and there was swelling of lymph nodes in the right axillary. Attention was provided in light of the potential for CSD. Nevertheless, the patient exhibited visible swelling in the anal region during the disease, accompanied by fever symptoms. As a result, the possibility of perianal abscess and other conditions could not be ruled out, and it remained uncertain whether the cat bite directly caused the fever. Therefore, no medication was given on the first day, and ancillary examinations and anorectal consultation were arranged to further assist in the diagnosis. The results of the main positive ancillary examinations after admission are shown in **Table 1**. Cellular and humoral immunity analysis indicated hypo-immunity (see **Table 2**) and hepatitis B surface antigen positivity. The findings of other laboratory tests, including complete results such as complete blood count, interleukin-6 (IL-6), blood biochemistry, coagulation tests, thyroid function, connective tissue disease-related antibodies, and tumor markers, were all within the normal range. Tests for human immunodeficiency virus (HIV), *Treponema pallidum*, *Mycobacterium tuberculosis*, *Mycoplasma pneumoniae*, and other viruses (e.g., cytomegalovirus, herpes simplex virus, coronavirus disease 2019 [COVID-19], rubella virus, influenza A virus, influenza B virus, respiratory syncytial virus, respiratory adenovirus, human rhinovirus) were negative, and D-glucan test and sputum culture showed no significant abnormalities.

**Table 1:** The temperature during hospitalization, the main positive lab indicators, and the treatment protocol.

Hospitalization days	body temperature	inflammatory indicators	Results	Reference range of normal value	treatment
Day 1	36.7°C	total number of WBC	4.47	3.5-9.5*10^9/L	without treatment
		the count of neutrophil	1.76 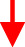	1.8-6.3*10^9/L	
		ESR	37 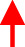	0-20mm/H	
		CRP	5.09	<10mg/L	
		PCT	0.07 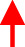	0-0.05ng/ml	
		IL-6	4.32	0.00-7.00pg/ml	
Day 2	39°C	total number of WBC	7.63	3.5-9.5*10^9/L	azithromycin 0.5g once a day
		the count of neutrophil	3.53	1.8-6.3*10^9/L	
		CRP	3.37	<10mg/L	
		PCT	0.09 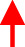	0-0.05ng/ml	
		IL-6	76.3 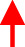	0.00-7.00pg/ml	
Day 3	40.4°C				azithromycin 0.25g once a day
Day 4	38.6°C				azithromycin 0.25g once a day+ doxycycline 0.1g twice a day
Day 5	36.9°C				the same as day 4
Day 6	37.5°C				the same as day 4
Day 7	36.6°C	the count of neutrophil	1.35 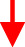	1.8-6.3*10^9/L	doxycycline 0.1g twice a day
		CRP	4.7mg/L	<10mg/L	
		PCT	0.10 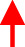	0-0.05ng/ml	
		IL-6	4.72	0.00-7.00pg/ml	
Day 8	36.7°C				the same as day 7
Day 9	36.8°C				the same as day 7
Day 10	36.3°C				the same as day 7

WBC, White Blood Cell; CRP, C-Reactive Protein; ESR, Erythrocyte Sedimentation Rate; PCT, Procalcitonin; IL-6, Interleukin-6. The red upward arrow indicates above normal value, while the red downward arrow indicates below normal value.

**Table 2:** Cellular and humoral immunity.

Cellular immunity		
Testing items	Results	Reference range of normal value
Counts of T lymphocyte	1033	763-2838cell/μl
Counts of CD4+T lymphocyte	131 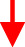	381-1610 cell/μl
Counts CD8+T lymphocyte	467	162-1165 cell/μl
Counts of NK cells	124	79-792 cell/μl
Counts B lymphocyte	38 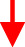	92-565 cell/μl
Percentage of T lymphocyte	85.62	55.49-87.49%
Percentage of CD4+T lymphocyte cells	10.84 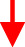	36.67-76.26%
Percentage of CD8+T lymphocyte cells	38.71	18.43-57.37%
CD4/CD8 ratio	0.28 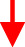	0.66-3.62%
Percentage of NK cells	10.3	4.23-32.02%
Percentage of B lymphocyte cells	3.19 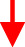	7.12-20.71%
humoral immunity		
Testing items	Results	Reference range of normal value
Immunoglobulin G	17.44 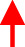	7-16g/L
Immunoglobulin A	2.23	0.7-4g/L
Immunoglobulin M	1.53	0.4-2.3g/L
Immunoglobulin E	142 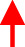	<100KIU/L
Complement C3	0.805 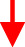	0.82-1.80g/L
Complement C4	0.282	0.1-0.4g/L

The red upward arrow indicates above normal value, while the red downward arrow indicates below normal value.

Superficial lymph node ultrasound showed right supraclavicular fossa lymphadenectasis, right axillary lymphadenectasis with structural abnormality, and left axillary lymphadenectasis with no structural abnormality. Lymph nodes in the bilateral cervical and bilateral inguinal were found without any structural abnormality. The chest and abdomen computerized tomography (CT) scan detected many tiny nodules in the upper and lower lobes of both lungs, These nodules are perhaps indicative of inflammation, and further examination was advised. Several stripe lesions were detected in the right middle and left lower lobes of the lungs. There was a visible thickening in the left pleural region. Enlarged lymph nodes were identified in the right axilla, and further evaluation was recommended. A number of small cysts were discovered in the liver. Both kidneys exhibited calcium salt accumulation; small cysts were discovered in the right kidney, and the sigmoid colon was lengthy. No substantial abnormalities were observed in electrocardiogram, heart ultrasound, and rectal ultrasound. The anorectal physical examination revealed no abnormalities. No specific intervention was recommended.

A CSD diagnosis was suggested on the second day of hospitalization based on the results that were presented. The patient’s primary symptom was lymphadenopathy. According to the Sanford Guide, azithromycin (0.5 g once a day for 1 day; 0.25 g once a day for 4 days) was prescribed, but during the treatment, the patient continued to have a fever as high as 40.4°C; the treatment was ineffective. It was determined to perform a needle biopsy of the lymph nodes in order to confirm the diagnosis. The patient declined to have the puncture because they believed that the intrusive procedure would increase the danger of infection spreading and delay healing of the puncture site. Hence, mNGS was performed. *B. henselae* (sequence number: 3, relative abundance = 100%), indicating bacteremia, was detected by mNGS. As per the Sanford Guide, the patient received a dose of 100 mg of doxycycline administered twice daily. Following the therapy, the maximum body temperature is reduced in comparison to the value taken prior to the treatment and the restored to its normal level over time.

**Figure 3:**
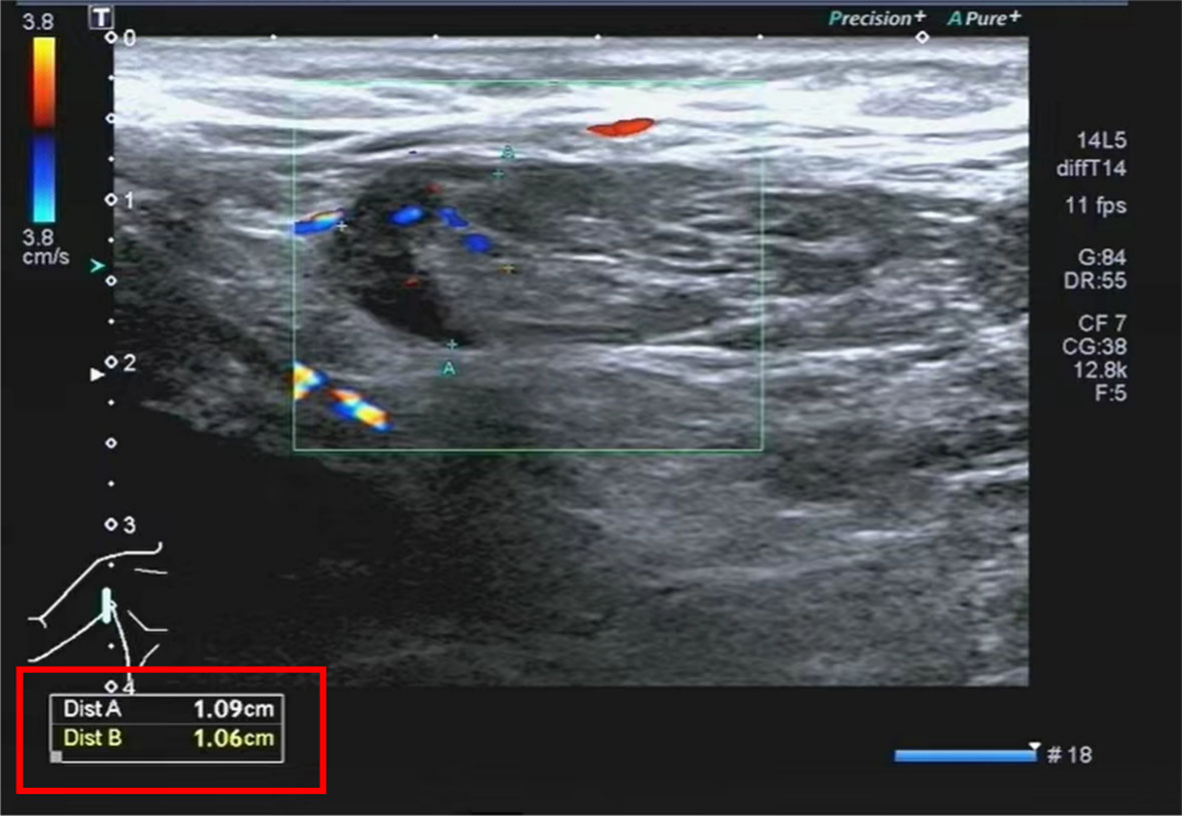
Lymph nodes in the right axilla under Color Doppler ultrasound.

The lymph nodes underwent re-examination and presented a reduction in size. Therefore, the patient was released from the hospital and proceeded to administer doxycycline orally for 2 weeks. During the 2-week follow-up, the patient’s overall condition was satisfactory, and he did not report any discomfort. The right axillary lymph nodes were re-examined and had shrunk (see **Figure 3**) as compared to the time of admission (the patient temperature during hospitalization, the main positive lab indicators, the size of the right axillary lymph node, and the treatment protocol are shown in **Table 1**).

## 3 Metagenomic next-generation sequencing

Blood samples obtained into 10-mL Vacuum blood collection tubes (Kang Jian, CHN) were centrifuged to remove cells for reduction of the host-background nucleic acids. Afterward, cell-free DNA (cf DNA) was extracted using a Magnetic Serum/Plasma DNA Maxi Kit (TIANGEN, China) according to the instructions provided by the manufacturer. DNA extraction yield was quantified using a QuantiT dsDNA HS Assay Kit and Qubit 3.0 Fluorometer (Thermo Scientific, USA). Enzymatic shearing was performed for the fragmentation (~200 bp) of DNA molecules, and libraries were constructed using the Nextera XT DNA Library Preparation Kit (Illumina, USA). The quality of the prepared libraries was assessed by a 2100 Bioanalyzer using the High Sensitivity DNA Assay (Agilent Technologies, USA). Metagenome shotgun sequencing was performed in a single-end 75-bp mode using the NextSeq 500/550 High Output Kit (92 cycles) on an Illumina NextSeq 550 Dx sequencer. No-Template Control (NTC) samples were sequenced concurrently to evaluate potential contamination during the wet lab studies.

## 4 Bioinformatic analysis of species-level abundance profiling

The initial raw sequencing data underwent a quality control procedure using Trimmomatic v0.36 to eliminate adaptor sequences, eliminate low-quality tails, and remove subpar reads. Subsequently, the short-read alignment tool Bowtie v2.2.6 was utilized to eliminate the reads that were aligned to the human reference genome GRCh37. Read duplication was then performed using in-house scripts. Taxonomic classification of microbial reads was conducted using Kraken v2.0.9-beta and a custom k-mer database, which was constructed using 51,543 genomes of about 27,000 species from the National Center for Biotechnology Information (NCBI) assembly databases.

## 5 Discussion

Long latency is an indicator of CSD, and during a consultation, patients typically do not voluntarily disclose a history of cat scratches or bites. Furthermore, it might be difficult to diagnose CSD based alone on abnormal physical symptoms and clinical manifestation because the primary skin lesions may have healed by the time of the consultation. Diagnosis of this condition is quite challenging and relies mostly on the patient’s history of cat bite or exposure, the presence of primary skin lesions, the occurrence of fever, lymphadenectasis, and the results obtained from *in vitro* culture or DNA amplification procedures (Chen et al., 2007). Margileth et al. reported that the diagnostic criteria for CSD mainly comprise the four aspects described in **Table 3** (Margileth, 2000). In addition, the CSD antigen skin test can serve as an important diagnostic tool (Margileth, 1993; Margileth and Baehren, 1998). However, due to variations across patients, changes in clinical presentation, and the reliability of laboratory testing, it is challenging to diagnose a condition by strictly adhering to the diagnostic criteria presented in **Table 3**.

**Table 3:** Diagnosis of cat scratch disease.

Criteria *
1. Cat or flea contact with or without a scratch mark or a regional inoculation lesion (skin papule, eye granuloma, mucous membrane).
2. Laboratory/radiology: negative purified protein derivative or serology for other infectious causes of adenopathy; sterile pus aspirated from node, polymerase chain reaction assay positive; *Bartonella henselae, Bartonella quintana*, or Afipia felis: highest sensitivity. CT scan: liver/spleen abscesses
3. Positive enzyme immunoassay or indirect fluorescent antibody assay serology test >1:64 for *B. henselae* or *B. quintana* or *Bartonella clarridgeiae*; fourfold rise in titer between acute and convalescent specimens is definitive.
4. Biopsy of node, skin, liver, bone, or eye granuloma showing granulomatous inflammation compatible with cat-scratch disease; positive Warthin-Starry silver stain

*Three of four criteria confirm the diagnosis; in an atypical case all four criteria may be needed.

mNGS can simultaneously detect multiple pathogens in one sample, and serve as a suitable technique for detection of rare, atypical, and complicated infections (Miller et al., 2018). Results for mNGS can be acquired on the same day (24 h). The results show that when compared to laboratory culture and empirical adjustment, antibacterial agent dose modification may substantially decrease the length of time patients spend in the intensive care unit (ICU) (Xie et al., 2019). Studies have shown that the sensitivity of mNGS is more effective than that of the conventional culture approach. This technique provides substantial advantages in the detection of fungi, tuberculosis (TB), viruses, and anaerobic bacteria (Miao et al., 2018). Wang et al. found that mNGS was more sensitive than the traditional culture method in the detection of bacteria and fungi in transbronchial lung biopsy (TBLB), bronchoalveolar lavage fluid (BALF), and bronchial brush (BB) specimens (Wang et al., 2020). Previous research found no indications of infection based on clinical symptoms and laboratory examinations. However, the evaluation of lymph node biopsy samples indicated a possible case of CSD; Further analysis using mNGS confirmed the presence of *B. henselae* in the lymph nodes (Yang et al., 2020). In addition, visceral leishmaniasis (Chen H. et al., 2020), psittacosis (Chen X. et al., 2020), and Seoul virus (Xie et al., 2021) have been detected using mNGS. Qian et al. suggest that the prompt application of mNGS may be helpful in diagnosing suspected infections caused by a combination of different or uncommon pathogens. This is particularly important for persons with weakened immune system or those who are critically ill and in need of immediate medical intervention (Qian et al., 2020). Although we had a suspicion that the patient was experiencing CSD upon admission to the hospital, our medical facility did not conduct targeted serological and PCR tests and the patient declined to undergo a lymph node biopsy. mNGS was performed using the peripheral blood sample, and *B. henselae* was detected at about 24 h, which further confirmed the diagnosis. The clinical symptoms resolved after relevant treatment for CSD, which in turn verified the results of mNGS.

Although mNGS offers substantial advantages in quickly identifying infections, the interpretation of mNGS results can be quite complex. Currently, there is no established criterion for interpreting mNGS results. It is necessary to consider the quality of the specimen, the quality of the testing technique, the rarity of the pathogen, and its relative and absolute abundance when interpreting mNGS results (Gu et al., 2019). A difficult task is typically to determine if the identified pathogens are colonizing bacteria, background bacteria, or pathogenic bacteria. Therefore, it is crucial to systematically examine, interpret, and validate laboratory tests and imaging studies (Nan et al., 2022). Obtaining specimens and conducting additional testing such as biopsy and serologic tests are crucial, especially when detecting uncommon pathogens (Chiu and Miller, 2019). Additionally, mNGS can offer minimal information regarding antibiotic susceptibility, a crucial factor in determining decisions for antibacterial therapy.

The treatment of CSD varies depending on the clinical manifestation and immunity of the patient (Mazur-Melewska et al., 2015). Indeed, CSD is self-limiting in typical patients with normal immunity and may not require antibiotic treatment, although follow-up is needed. For instances of lymph node suppuration, it is advisable to perform puncture and drainage rather than incision and drainage. When performing a puncture, it is important to move the needle into several positions, as the microabscess typically has many compartments (Margileth, 2000; Rolain et al., 2004). In adult patients with severe lymphadenopathy, administration of 10 mg/kg azithromycin on day 1 and 5 mg/kg azithromycin daily from day 2 to 5 is an effective treatment (Mazur-Melewska et al., 2015). Among patients with asymptomatic bacteremia, 100 mg of doxycycline twice a day for 15 days is effective. CSD can cause ophthalmopathy, osteomyelitis, encephalopathy, endocarditis, and bacterial hemangioma if not treated promptly with antimicrobial agents and/or surgical intervention. In such cases, CSD can be fatal and can spread to other organ systems. The preferred drugs for CSD are azithromycin, erythromycin, rifampicin, and doxycycline (Mazur-Melewska et al., 2015; Uluğ, 2015), which are often prescribed in combination. *In vitro*, *Bartonella* is sensitive to many antimicrobial agents (*e.g*., macrolides, aminoglycosides, β-lactams, third-generation cephalosporins, trimethoprim sulfamethoxazole, rifampicin, and ciprofloxacin) (Ives et al., 1997). *B. henselae* is an intracellular parasite that can grow in erythrocytes or endothelial cells of the host. Many antibiotics cannot reach *Bartonella* due to their weak cell membrane permeability, resulting in the persistence of the bacteria in the host bloodstream. Therefore, bactericidal agents that traverse cell membranes, including erythromycin, doxycycline, azithromycin, and rifampicin are favored for therapeutic purposes.

After using azithromycin, the patient experienced a high fever in the evening accompanied by an increase in IL-6. While maintaining vigilance regarding the potential Jarisch-Herschel’s reaction, the patient manifested considerable pain in the primary skin lesion located at the fingertip when he was experiencing fever. This pain spread to the right armpit via the lymphatic. No muscle pain, headache, fluctuations in circulating neutrophil count, or changes in blood pressure were reported. Consequently, we prioritized the entry of bacteria into the bloodstream from the location rather than Jarisch-Herschel’s reaction. The findings of mNGS further corroborated our hypothesis.

The patient did not have a fever on the day of admission, and the test results only indicated mild inflammation. Additional tests revealed no unusual, and no treatment was recommended. Lymphadenectasis was suspected based on the findings of the superficial lymph node ultrasound, and the patient disclosed a prior incident of cat bites. With lymphadenopathy as the main manifestation, the patient was subjected to CSD diagnosis and azithromycin (0.5 g once a day for 1 day, 0.25 g once a day for 4 days) was given for treatment. High fever appeared after the first day of the treatment process. Due to his compromised immune system, it was crucial to remain vigilant for the worsening of his condition and the occurrence of widespread CSD. mNGS was utilized to efficiently identify the underlying cause of the disease by analyzing a sample peripheral bold, which revealed the presence of *B. henselae* bacteremia in the bloodstream. According to the Chinese “practical internal medicine”, the antibiotics were adjusted to azithromycin + doxycycline, which led to a gradual decrease in the body temperature. The patient was released from the hospital but continued a regimen of doxycycline for 2 weeks. The patient’s condition attained a stable state, and there were no more cases of recurrence during the follow-up period.

## 6 Conclusion

CSD has been misdiagnosed and missed diagnosed in various cases for the following reasons: (1) It has a long latency (2 weeks to several months), and the patient may not mention the history of cat or dog scratches at the time of consultation. The primary skin lesions may have healed and only presented with enlarged lymph nodes or fever, causing clinicians to oversee the possibility of CSD. (2) The occurrence rate is minimal, and the clinical manifestations are uncommon, as fever and lymphadenectasis might be incorrectly identified as reactive hyperplasia of lymph nodes, tuberculous lymphadenitis, or lymph node metastases. (3) The sensitivity and specificity of serologic tests for *B. henselae* are variable, and other detection methods, such as culture and Warthin-Starry silver staining have low sensitivity and specificity. The use of PCR and immunohistochemistry is limited in routine diagnostic laboratories. mNGS aims to identify pathogens rapidly and accurately and can serve as an auxiliary technique for the diagnosis of CSD. Nevertheless, it is essential to validate the application and findings of mNGS by cross-referencing them with clinical data in order to prevent inaccurate diagnoses resulting from firmly depending on the evidence provided by a solitary screening method.

## Data availability statement

The data presented in the study are deposited in the National Center for Biotechnology Information repository, accession number PRJNA1045726.

## Ethics statement

The studies involving humans were approved by Ethics Committee for Scientific Research and Clinical Trials of the Third People’s Hospital of Chengdu. The studies were conducted in accordance with the local legislation and institutional requirements. The human samples used in this study were acquired from clinical data of patients during hospitalization. Written informed consent for participation was not required from the participants or the participants’ legal guardians/next of kin in accordance with the national legislation and institutional requirements. Written informed consent was obtained from the individual(s) for the publication of any potentially identifiable images or data included in this article. Written informed consent was obtained from the participant/patient(s) for the publication of this case report.

## Author contributions

TZ: Conceptualization, Data curation, Formal analysis, Methodology, Writing – original draft, Writing – review & editing. YZ: Conceptualization, Writing – review & editing. HZ: Conceptualization, Writing – review & editing. YL: Project administration, Conceptualization, Writing – review & editing.

## References

[B1] Chen H. Fan C. Gao H. Yin Y. Wang X. Zhang Y.. (2020). Leishmaniasis diagnosis via metagenomic next-generation sequencing. Front. Cell. Infect. Microbiol. 10. doi: 10.3389/fcimb.2020.528884 PMC7538539 33072623

[B3] Chen X. Cao K. Wei Y. Qian Y. Liang J. Dong D.. (2020). Metagenomic next-generation sequencing in the diagnosis of severe pneumonias caused by Chlamydia psittaci. Infection 48 (4), 535–542. doi: 10.1007/s15010-020-01429-0 32314307 PMC7223968

[B2] Chen T. C. Lin W. R. Lu P. L. Lin C. Y. Chen Y. H. (2007). Cat scratch disease from a domestic dog. J. Formos. Med. Assoc. 106 (2 Suppl), S65–S68. doi: 10.1016/s0929-6646(09)60356-9 17493900

[B4] Chiu C. Y. Miller S. A. (2019). Clinical metagenomics. Nat. Rev. Genet. 20 (6), 341–355. doi: 10.1038/s41576-019-0113-7 30918369 PMC6858796

[B5] Chomel B. B. (2000). Cat-scratch disease. Rev. Sci. Tech. 19 (1), 136–150. doi: 10.20506/rst.19.1.1204 11189710

[B6] Cotté V. Bonnet S. Le Rhun D. Le Naour E. Chauvin A. Boulouis H. J.. (2008). Transmission of Bartonella henselae by Ixodes ricinus. Emerg. Infect. Dis. 14 (7), 1074–1080. doi: 10.3201/eid1407.071110 18598628 PMC2600320

[B7] Dornbos D. Morin J. Watson J. R. Pindrik J. (2016). Thoracic osteomyelitis and epidural abscess formation due to cat scratch disease: case report. J. Neurosurg. Pediatr. 25 (6), 713–716. doi: 10.3171/2016.7.Peds1677 27662446

[B8] Florin T. A. Zaoutis T. E. Zaoutis L. B. (2008). Beyond cat scratch disease: widening spectrum of Bartonella henselae infection. Pediatrics 121 (5), e1413–e1425. doi: 10.1542/peds.2007-1897 18443019

[B9] Gamblin T. C. Nobles-James C. Bradley R. A. Katner H. P. Dale P. S. (2005). Cat scratch disease presenting as breast mastitis. Can. J. Surg. 48 (3), 254–255. 16013636 PMC3211541

[B10] Gu W. Miller S. Chiu C. Y. (2019). Clinical metagenomic next-generation sequencing for pathogen detection. Annu. Rev. Pathol. 14, 319–338. doi: 10.1146/annurev-pathmechdis-012418-012751 30355154 PMC6345613

[B11] Hong H. Li T. Ying Y. An Q. Liu H. Liang K. (2023). Cat-scratch disease manifesting as uveitis and binocular fundus nodular lesions: a case report. BMC Ophthalmol. 23 (1), 345. doi: 10.1186/s12886-023-03063-4 37544996 PMC10405493

[B12] Ives T. J. Manzewitsch P. Regnery R. L. Butts J. D. Kebede M. (1997). *In vitro* susceptibilities of Bartonella henselae, B. quintana, B. elizabethae, Rickettsia rickettsii, R. conorii, R. akari and R. prowazekii to macrolide antibiotics as determined by immunofluorescent-antibody analysis of infected Vero cell monolayers. Antimicrob. Agents Chemother. 41 (3), 578–582. doi: 10.1128/aac.41.3.578 9055996 PMC163754

[B14] Johnson A. (2020). Ocular complications of cat scratch disease. Br. J. Ophthalmol. 104 (12), 1640–1646. doi: 10.1136/bjophthalmol-2019-315239 32122915

[B13] Juan H. Gan D. L. (2011). Research progress in clinical pathology of cat scratch disease. Clin. Exp. Pathol. 27 (03), 293–297. doi: 10.13315/j.cnki.cjcep.2011.03.005

[B15] Maman E. Bickels J. Ephros M. Paran D. Comaneshter D. Metzkor-Cotter E.. (2007). Musculoskeletal manifestations of cat scratch disease. Clin. Infect. Dis. 45 (12), 1535–1540. doi: 10.1086/523587 18190312

[B16] Margileth A. M. (1993). Cat scratch disease. Adv. Pediatr. Infect. Dis. 8, 1–21. 8216999

[B17] Margileth A. M. (2000). Recent advances in diagnosis and treatment of cat scratch disease. Curr. Infect. Dis. Rep. 2 (2), 141–146. doi: 10.1007/s11908-000-0026-8 11095849

[B18] Margileth A. M. Baehren D. F. (1998). Chest-wall abscess due to cat-scratch disease (CSD) in an adult with antibodies to Bartonella clarridgeiae: case report and review of the thoracopulmonary manifestations of CSD. Clin. Infect. Dis. 27 (2), 353–357. doi: 10.1086/514671 9709886

[B19] Mazur-Melewska K. Mania A. Kemnitz P. Figlerowicz M. (2015). Cat-scratch disease: a wide spectrum of clinical pictures. Postepy dermatologii i alergologii 32 (3), 216–220. doi: 10.5114/pdia.2014.44014 26161064 PMC4495109

[B20] Miao Q. Ma Y. Wang Q. Pan J. Zhang Y. Jin W.. (2018). Microbiological diagnostic performance of metagenomic next-generation sequencing when applied to clinical practice. Clin. Infect. Dis. 67 (suppl_2), S231–s240. doi: 10.1093/cid/ciy693 30423048

[B21] Miller J. M. Binnicker M. J. Campbell S. Carroll K. C. Chapin K. C. Gilligan P. H.. (2018). A guide to utilization of the microbiology laboratory for diagnosis of infectious diseases: 2018 update by the infectious diseases society of America and the American society for microbiology. Clin. Infect. Dis. 67 (6), e1–e94. doi: 10.1093/cid/ciy381 29955859 PMC7108105

[B22] Nan X. Zhang Y. Su N. Yang L. Pan G. (2022). Application value of metagenomic next-generation sequencing for bloodstream infections in pediatric patients under intensive care. Infect. Drug Resist. 15, 1911–1920. doi: 10.2147/idr.S357162 35465251 PMC9031986

[B23] Qian Y. Y. Wang H. Y. Zhou Y. Zhang H. C. Zhu Y. M. Zhou X.. (2020). Improving pulmonary infection diagnosis with metagenomic next generation sequencing. Front. Cell. Infect. Microbiol. 10. doi: 10.3389/fcimb.2020.567615 PMC7874146 33585263

[B24] Rolain J. M. Brouqui P. Koehler J. E. Maguina C. Dolan M. J. Raoult D. (2004). Recommendations for treatment of human infections caused by Bartonella species. Antimicrob. Agents Chemother. 48 (6), 1921–1933. doi: 10.1128/aac.48.6.1921-1933.2004 15155180 PMC415619

[B25] Schutze G. E. (2000). Diagnosis and treatment of Bartonella henselae infections. Pediatr. Infect. Dis. J. 19 (12), 1185–1187. doi: 10.1097/00006454-200012000-00014 11144381

[B26] Tey M. S. Govindasamy G. Vendargon F. M. (2020). The clinical spectrum of ocular bartonellosis: a retrospective study at a tertiary centre in Malaysia. J. Ophthalmic Inflamm. Infect. 10 (1), 31. doi: 10.1186/s12348-020-00224-0 33191467 PMC7667203

[B27] Uluğ M. (2015). Evaluation of cat scratch disease cases reported from Turkey between 1996 and 2013 and review of the literature. Cent. Eur. J. Public Health 23 (2), 170–175. doi: 10.21101/cejph.a4040 26851430

[B28] Wang Q. Wu B. Yang D. Yang C. Jin Z. Cao J.. (2020). Optimal specimen type for accurate diagnosis of infectious peripheral pulmonary lesions by mNGS. BMC Pulm. Med. 20 (1), 268. doi: 10.1186/s12890-020-01298-1 33059646 PMC7566056

[B29] Windsor J. J. (2001). Cat-scratch disease: epidemiology, aetiology and treatment. Br. J. Biomed. Sci. 58 (2), 101–110. 11440202

[B31] Xie Y. Du J. Jin W. Teng X. Cheng R. Huang P.. (2019). Next generation sequencing for diagnosis of severe pneumonia: China 2010-2018. J. Infect. 78 (2), 158–169. doi: 10.1016/j.jinf.2018.09.004 30237069

[B30] Xie D. Xu W. Xian Y. Yuan X. Huang Z. You J.. (2021). Rare case of intracranial hemorrhage associated with Seoul virus infection diagnosed by metagenomic next-generation sequencing. J. Clin. Lab. Anal. 35 (2), e23616. doi: 10.1002/jcla.23616 33084078 PMC7891533

[B32] Yang T. Mei Q. Zhang L. Chen Z. Zhu C. Fang X.. (2020). Hemophagocytic lymphohistiocytosis is associated with Bartonella henselae infection in a patient with multiple susceptibility genes. Ann. Clin. Microbiol. Antimicrob. 19 (1), 28. doi: 10.1186/s12941-020-00370-2 32517705 PMC7281694

